# Aerobic Exercise Alleviates Abnormal Autophagy in Brain Cells of APP/PS1 Mice by Upregulating AdipoR1 Levels

**DOI:** 10.3390/ijms23179921

**Published:** 2022-08-31

**Authors:** Ye Jian, Shunling Yuan, Jialun Yang, Yong Lei, Xuan Li, Wenfeng Liu

**Affiliations:** 1 Hunan Provincial Key Laboratory of Physical Fitness and Sports Rehabilitation, Hunan Normal University, Changsha 410012, China; 2 Key Laboratory of Protein Chemistry and Developmental Biology of Ministry of Education, Hunan Normal University, Changsha 410081, China

**Keywords:** exercise, Alzheimer’s disease, adiponectin, AdipoR1, autophagy

## Abstract

Abnormalities in autophagy are associated with Alzheimer’s disease (AD)-like lesions. Studies have shown that exercise can significantly improve AD autophagy abnormalities, but the mechanism underlying this phenomenon remains unclear. APN not only has an important regulatory effect on AD autophagy abnormalities, but also is affected by exercise. Therefore, this study aims to reveal the pathway by which exercise regulates abnormal autophagy in AD using the APN–AdipoR1 signaling pathway as an entry point. The results of the study showed that APP/PS1 double transgenic AD model mice (24 weeks) showed decreased AdipoR1 levels in the brain, abnormal autophagy, increased Aβ deposition, and increased cell apoptosis, and dendritic spines and cognitive function were reduced. Twelve weeks of aerobic exercise enhanced lysosomes and alleviated abnormal autophagy by activating the AdipoR1/AMPK/TFEB signaling pathway in the brains of AD mice, thereby alleviating Aβ deposition and its associated AD-like abnormalities. These findings suggest that the AdipoR1 plays an important role in aerobic exercise’s alleviation of abnormal autophagy in AD brain cells and alleviation of AD-like lesions.

## 1. Introduction

Alzheimer’s disease (AD) is a neurodegenerative disease commonly referred to as senile dementia. The amyloid hypothesis states that the abnormal accumulation of amyloid β-protein (Aβ), a transmembrane amyloid precursor proteolytic fragment, in the brain is the main cause of AD [1]. Autophagy is an effective way to clear abnormal proteins such as Aβ in the brain, and abnormal autophagy in AD aggravates the disease [2]. Therefore, the regulation of autophagy is a possible approach to the treatment of AD [3]. Existing studies have shown that exercise can promote the degradation of Aβ by regulating autophagy in AD brain cells, thereby improving cognitive impairment [4,5,6]. However, its specific pathway and mechanism remain unclear.

Adiponectin (APN) is a protein hormone secreted by adipose tissue and has neuroprotective effects [7]. The main types of adiponectin receptors (AdipoR) characterized in vivo are AdipoR1, AdipoR2, and T-cadherin; AdipoR1 and AdipoR2 are the main receptors for APN and are widely expressed in various brain regions such as the cerebral cortex, nucleus basalis of Meynert, and hippocampus [7]. AdipoR1 activates the adenosine 5′-monophosphate (AMP)-activated protein kinase (AMPK) pathway, and AdipoR2 activates the peroxisome proliferator-activated receptor-α pathway [7]. APN mediates the protective effect of exercise on the central nervous system. Studies have shown that exercise can promote the expression of APN and its receptors [8,9,10]. Many experimental studies of APN knockout have shown that APN is essential for exercise’s alleviation of brain damage and cognitive dysfunction caused by chronic diseases [11,12,13,14], and APN plays an important role in alleviating AD. Studies have shown that APN can alleviate AD by reducing the neuronal damage caused by the disease [15,16,17]. This neuroprotective effect of APN is achieved, to a certain extent, by the upregulation of the AdipoR1/AMPK signaling pathway, activating autophagy [16,17,18,19]. This provides a new perspective on how exercise regulates abnormal autophagy in AD brain cells. Therefore, does exercise activate autophagy in brain cells by upregulating the APN–AdipoR1 signaling pathway, thereby alleviating AD?

In this study, APPswe/PSEN1dE9 (APP/PS1) double transgenic AD model mice were used as experimental objects. The effects of 12 weeks’ aerobic treadmill exercise on learning and memory ability, brain amyloid plaques, neuronal apoptosis, dendritic spine density, serum APN levels, brain APN levels, brain AdipoR1 levels, and AMPK in APP/PS1 mice, as well as the effect of autophagy-related proteins in brain cells were observed to determine the role of the APN–AdipoR1 signaling pathway in exercise’s regulation of AD autophagy abnormalities. At the same time, the expression levels of transcription factor EB (TFEB) and nuclear translocation in brain cells, the expression levels of lysosomal function-related proteins and genes downstream of TFEB, and the expression levels of the phospholipase C (PLC)/downstream regulatory element antagonist modulator (DREAM/Kcnip3)/E3 ligase Midline-1 (Mid1)/protein phosphatase 2A (PP2A) genes in the upstream signaling pathway for TFEB were detected to determine the possible role of the APN–AdipoR1 signaling pathway in exercise’s enhancement of AD lysosomal function.

## 2. Results

### 2.1. Exercise Alleviates Cognitive Impairment in AD Mice

**Figure 1:**
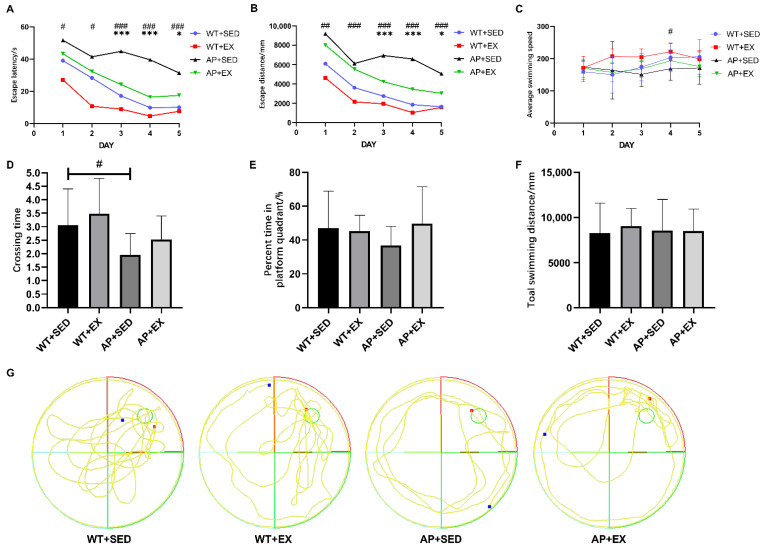
Twelve-week aerobic exercise alleviated cognitive impairment in AD mice. (**A**) Escape latency time during the learning phase in the MWM water maze test. (**B**) Escape latency swimming distance during the learning phase. (**C**) Average swimming speed during the learning phase. (**D**) Number of platform crossings during the retention phase. (**E**) Target quadrant time percentage for retention phase. (**F**) Total swimming distance during the retention phase. (**G**) Swimming path trajectory of the retention phase. The red square is the starting position, the blue square is the final position, and the yellow connection between them is the swimming path trajectory. The area enclosed by the red line is the first quadrant, which is the second quadrant, the third quadrant, and the fourth quadrant in counterclockwise order. The green circle in the first quadrant is the platform. *n* = 10 per group. Data are presented as means ± SDs. # *p* < 0.05, ## *p* < 0.01, and ### *p* < 0.001 vs. WT + SED mice; * *p* < 0.05, and *** *p* < 0.001 vs. AP + SED mice.

Aerobic exercise is considered to be an effective means of alleviating memory loss and cognitive dysfunction in AD patients [20]. Considered the test of choice for assessing learning and memory in basic and regulatory research, the Morris water maze (MWM) provides accurate and reproducible testing of reference memory, spatial working memory, and learning ability, and benefits include less training, no food deprivation, fast learning, no odor cues, etc. [21]. Therefore, our group used MWM to evaluate the effect of aerobic exercise on cognitive function in AD mice. The experimental results of the MWM learning phase (Figure 1A,B) showed that the average escape latency and swimming distance of the four groups of mice gradually shortened within five training days. Compared with the mice in the WT + SED group, the mice in the AP + SED group showed longer escape latencies and swimming distances during the five days’ training (*p* < 0.05). Aerobic exercise significantly decreased the escape latencies and swimming distances of the mice in the AP + EX group on days 3–5 compared to those in the AP + SED group (*p* < 0.05). The average swimming speed of the mice in the WT + SED group and the AP + SED group (Figure 1C) was only significantly different on the fourth day (*p* < 0.05), and there was no statistically significant difference between the AP + SED and AP + EX groups in the MWM learning phase (*p* > 0.05). The experimental results of the retention phase (Figure 1D,E) showed that compared with the mice in the WT + SED group, the mice in the AP + SED group had fewer platform crossings (*p* < 0.05). Compared with mice in the AP + SED group, aerobic exercise tended to increase the number of platform crossings and the percentage of time in the target quadrant of mice in the AP + EX group. In addition, there was no statistically significant difference in the total swimming distance (Figure 1F) among the four groups of mice (*p* > 0.05). The above results suggest that aerobic exercise can effectively alleviate the spatial learning and memory impairment of APP/PS1 mice without affecting the swimming ability of mice.

### 2.2. Exercise Reduces Aβ Accumulation in the Brains of AD Mice

**Figure 2:**
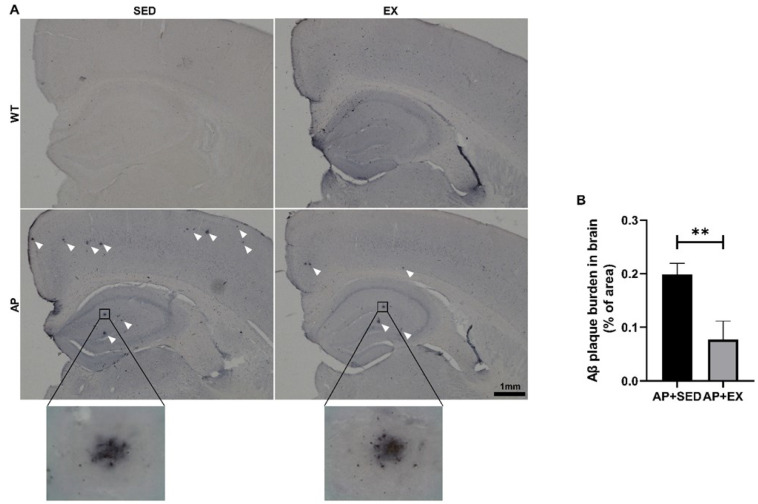
Exercise reduced brain Aβ plaques in AD mice. (**A**) Representative images of IHC of Aβ plaques in mouse brain tissue. The white arrows point to Aβ plaques. Scale bar: 1 mm. (**B**) The percentage of Aβ plaque area in mouse brain tissue was quantified to compare the Aβ content in brain tissue. *n* = 3 per group. Data are presented as means ± SDs. ** *p* < 0.01 vs. AP + SED mice.

Aβ is considered to be a key factor leading to memory and cognitive impairment in AD patients, and Aβ aggregates to form Aβ plaques, the main pathological feature of AD [1]. Therefore, our group analyzed the proportional Aβ plaque areas in mouse brain tissue by IHC. As shown in Figure 2B, compared with those for the mice in the AP + SED group, the proportional Aβ plaque areas in the brains of the AP + EX group were significantly reduced (*p* < 0.01).

### 2.3. Exercise Inhibits Brain Cell Apoptosis in AD Mice

**Figure 3:**
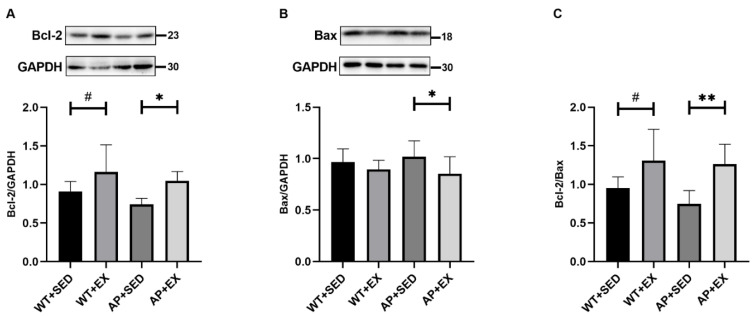
Exercise inhibited apoptosis in the brain cells of AD mice. Relative protein levels of (**A**) Bcl-2 and (**B**) Bax in brain tissue. (**C**) Bcl-2/Bax protein ratios in brain tissue. *n* = 6 per group. Data are presented as means ± SDs. # *p* < 0.05 vs. WT + SED mice, * *p* < 0.05, ** and *p* < 0.01 vs. AP + SED mice.

Aβ induces neuronal apoptosis in AD, leading to cognitive dysfunction [1]. In this study, we measured the antiapoptotic protein B-cell lymphoma-2 (Bcl-2), the proapoptotic protein Bcl-2-associated X protein (Bax), and their ratio to determine the apoptosis in the mouse brain. As shown in Figure 3A, aerobic exercise significantly increased the levels of the Bcl-2 protein in the brains of the mice in the WT + EX group compared to those in the WT + SED group (*p* < 0.05). Compared with mice in the AP + SED group, aerobic exercise significantly increased the levels of the Bcl-2 protein in the AP + EX group (*p* < 0.05). As shown in Figure 3B, compared with the AP + SED group, the Bax protein levels in the AP + EX group were significantly decreased (*p* < 0.05). As shown in Figure 3C, compared with the mice in the WT + SED group, aerobic exercise significantly increased the ratios of the Bcl-2/Bax proteins in the brains of the mice in the WT + EX group (*p* < 0.05). Compared with mice in the AP + SED group, aerobic exercise significantly increased the ratios of the Bcl-2/Bax proteins in the AP + EX group (*p* < 0.01). The above results suggest that aerobic exercise can effectively inhibit the apoptosis of brain cells in APP/PS1 mice.

### 2.4. Exercise Increases the Density of Dendritic Spines in AD Mice

**Figure 4:**
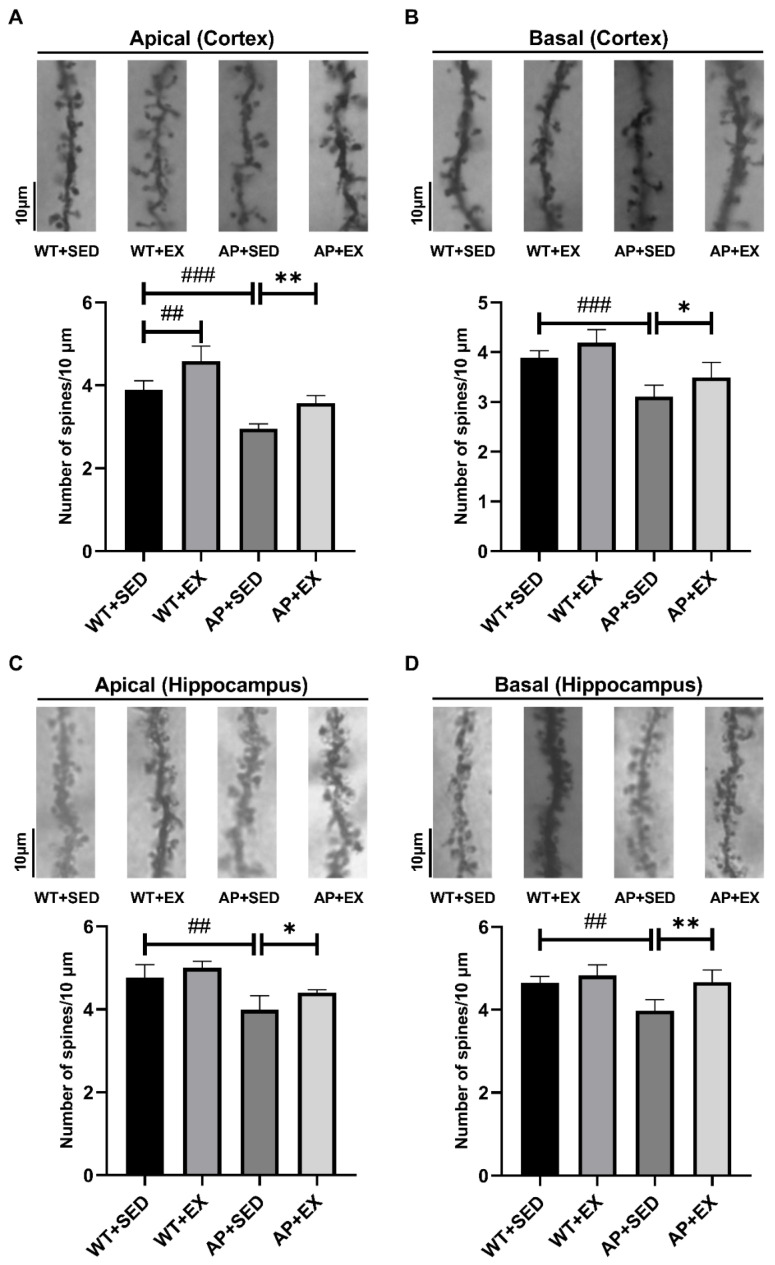
Exercise ameliorated the decrease in the dendritic spine density in the brain tissue of AD mice. (**A**–**D**) Representative images of Golgi staining of apical and basal dendritic spines in the cortices and hippocampi of mouse brain tissue and histograms showing the density of apical and basal dendritic spines in the cortex and hippocampus ((**A**) cortex apical; (**B**) cortex basal; (**C**) hippocampus apical; (**D**) hippocampus basal). Scale bar: 10 μm. *n* = 3–4 per group. Data are presented as means ± SDs. ## *p* < 0.01, ### *p* < 0.001 vs. WT + SED mice, * *p* < 0.05, and ** *p* < 0.01 vs. AP + SED mice.

Dendritic spines are spinous protrusions on the dendritic branches of nerve cells, and their reduced density is strongly correlated with cognitive impairment in AD [22]. This study quantified the density of dendritic spines in mouse brain tissue by Golgi staining. As shown in Figure 4A,B, compared with the mice in the WT + SED group, the density of apical and basal dendritic spines in the cortices of the mice in the AP + SED group was significantly reduced (*p* < 0.001). The density of apical dendritic spines in the cortices of the mice in the WT + EX group was significantly increased (*p* < 0.01). Compared with mice in the AP + SED group, aerobic exercise significantly increased the density of apical and basal dendritic spines in the cortices of the mice in the AP + EX group (apical, *p* < 0.01; basal, *p* < 0.05). As shown in Figure 4C,D, compared with the mice in the WT + SED group, the density of apical and basal dendritic spines in the hippocampi of the mice in the AP + SED group was significantly decreased (*p* < 0.01). Compared with the mice in the AP + SED group, aerobic exercise significantly increased the density of apical and basal dendritic spines in the hippocampi of the mice in the AP + EX group (apical, *p* < 0.05; basal, *p* < 0.01). The above results suggest that aerobic exercise can effectively increase the density of dendritic spines in the brain tissue of APP/PS1 mice.

### 2.5. Exercise Alleviates Abnormal Autophagy in the Brain Cells of AD Mice

**Figure 5:**
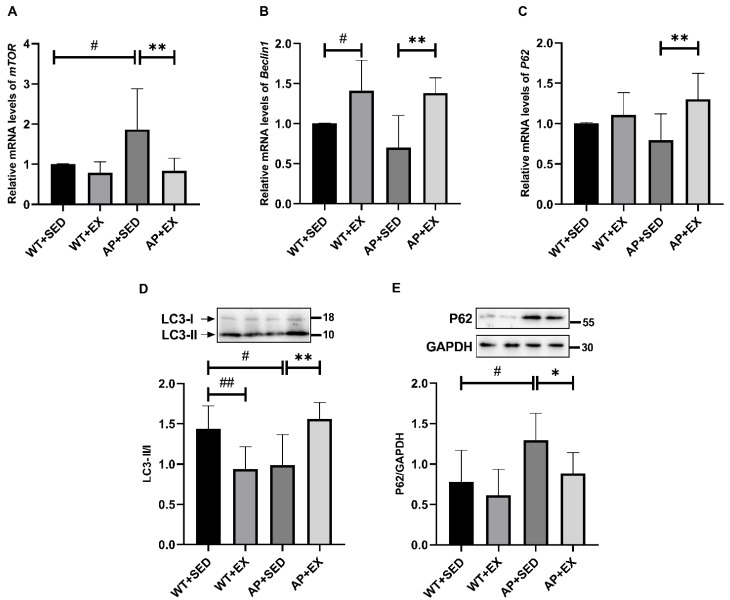
Exercise regulated autophagy in mouse brain tissue. The relative expression levels of (**A**) *mTOR*, (**B**) *Beclin1*, and (**C**) *P62* mRNA in brain tissue were measured by RT-PCR (*n* = 5). Relative protein levels of (**D**) LC3-II/I and (**E**) P62 in brain tissue (*n* = 6). Data are presented as means ± SDs. # *p* < 0.05, ## *p* < 0.01 vs. WT + SED mice, * *p* < 0.05, and ** *p* < 0.01 vs. AP + SED mice.

Autophagy plays an important role in alleviating AD-like lesions, Aβ accumulation, and dendritic spine loss in AD brain cells [2,23,24]. The mammalian target of rapamycin (mTOR) is a “master switch” for cellular synthesis and catabolism, and its overactivation is associated with autophagy inhibition in AD [25]. Therefore, our group detected the mRNA expression levels of *mTOR* and *Beclin1*, signaling molecules that regulate autophagy. As shown in Figure 5A, compared with that in the mice in the WT + SED group, the expression level of the *mTOR* mRNA in the AP + SED group was significantly increased (*p* < 0.05). Compared with the AP + SED group, aerobic exercise significantly downregulated the *mTOR* mRNA expression levels in the AP + EX group (*p* < 0.01). As a marker of autophagy initiation, Beclin1 is decreased in early AD and affects autophagy [26]. As shown in Figure 5B, compared with the mice in the WT + SED group, aerobic exercise significantly increased the expression level of the *Beclin1* mRNA in the brain tissue of the mice in the WT + EX group (*p* < 0.05). On the contrary, in the mice in the AP + SED group, the expression of the *Beclin1* mRNA showed a downward trend. Compared with the mice in the AP + SED group, aerobic exercise significantly increased the expression of the *Beclin1* mRNA in the brain tissue of the mice in the AP + EX group (*p* < 0.01). These results suggest that aerobic exercise may regulate abnormal autophagy in the brain cells of APP/PS1 mice.

In order to further study the effect of aerobic exercise on abnormal autophagy in AD, our group detected autophagy-related proteins, microtubule-associated protein 1 light chain 3 (LC3), and sequestosome-1 (P62). As a marker of autophagosome formation, LC3-II is associated with autophagic activity [27,28]. As shown in Figure 5D, compared with the mice in the WT + SED group, the LC3-II/I ratios in the brain cells of the mice in the WT + EX group and the AP + SED group were significantly decreased (WT + EX group, *p* < 0.01; AP + SED group, *p* < 0.05). Compared with the mice in the AP + SED group, aerobic exercise significantly increased the LC3-II/I ratios in the brain cells of the mice in the AP + EX group (*p* < 0.01). P62 localizes autophagic substrates to autophagosomes by interacting with LC3 and is degraded by autophagy together with autophagic substrates, so it can be used as an indicator of autophagic degradation [27,29]. Decreased levels of P62 are associated with autophagy activation [27,29]. As shown in Figure 5E, compared with the mice in the WT + SED group, the level of the P62 protein in the brain cells of the mice in the AP + SED group was significantly increased (*p* < 0.05). Compared with the mice in the AP + SED group, aerobic exercise significantly decreased the level of the P62 protein in the mice in the AP + EX group (*p* < 0.05). As shown in Figure 5C, there was no significant difference in the expression of the *P62* mRNA in the brain cells of the mice in the WT + EX group and AP + SED group, compared with the WT + SED group. Compared with the mice in the AP + SED group, the expression of the *P62* mRNA in the brain cells of the AP + EX group was significantly increased (*p* < 0.01). It was suggested that the difference in the expression level of the P62 protein among the groups was not caused by the difference in the transcription level of the *P62* gene, but was related to the difference in the level of autophagic degradation. The above results indicated that aerobic exercise alleviated the abnormal autophagy in the brain cells of APP/PS1 mice.

### 2.6. The Effect of Exercise on APN and Its Receptors in the Brains of AD Mice

**Figure 6:**
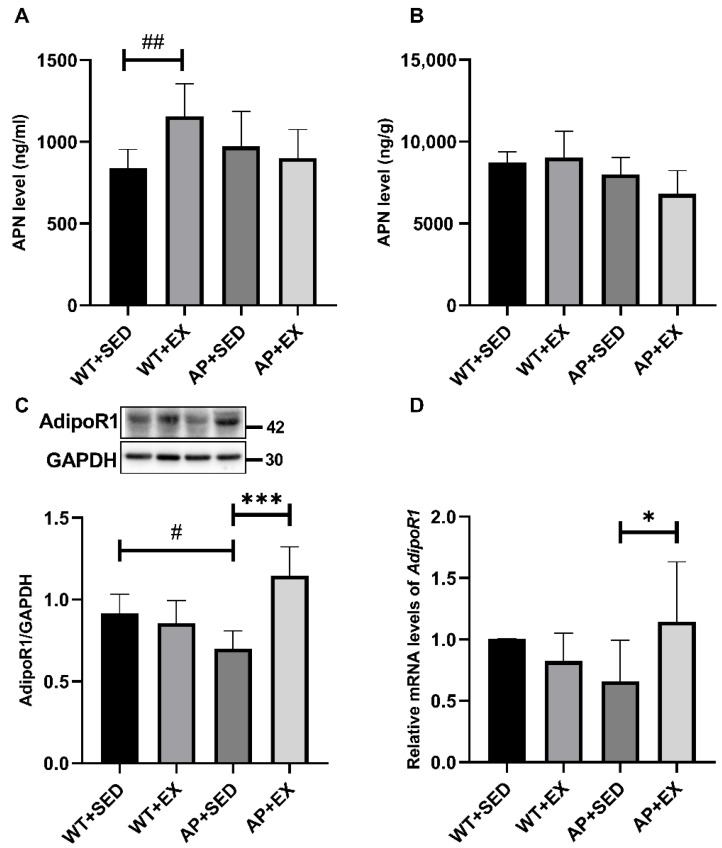
Exercise upregulates the AdipoR1 levels in the brains of AD mice. (**A**) Serum APN protein levels were measured by ELISA (*n* = 6). (**B**) Brain tissue APN protein levels were measured by ELISA (*n* = 4). (**C**) Relative protein levels of AdipoR1 in brain tissue (*n* = 6). (**D**) The relative expression of *AdipoR1* mRNA in brain tissue was measured by RT-PCR (*n* = 5). Data are presented as means ± SDs. # *p* < 0.05, ## *p* < 0.01 vs. WT + SED mice, * *p* < 0.05, and *** *p* < 0.001 vs. AP + SED mice.

APN is an adipokine secreted by adipose tissue and is a potential target for the remission of AD [7]. Exercise has been reported to induce increases in circulating APN and AdipoR1 levels [8,9,10]. However, whether aerobic exercise modulates APN and its receptors in the brains of AD mice has not been studied. The research group detected the serum and brain APN and brain AdipoR1 levels in AD mice. As shown in Figure 6A, compared with the mice in the WT + SED group, the serum APN protein levels of the mice in the WT + EX group were significantly increased (*p* < 0.01). As shown in Figure 6B, compared with the mice in the AP + SED group, the APN protein contents in the brains of the mice in the AP + EX group had a downward trend. As shown in Figure 6C, compared with the mice in the WT + SED group, the expression of the AdipoR1 protein in the brains of the mice in the AP + SED group was significantly decreased (*p* < 0.05). As shown in Figure 6C,D, compared with the mice in the AP + SED group, aerobic exercise increased the expression of the AdipoR1 protein and mRNA in the brains of the mice in the AP + EX group (protein, *p* < 0.001; mRNA, *p* < 0.05). As shown in Figure 6D, compared with the WT + SED group, the brain *AdipoR1* mRNA expression levels in the mice in the WT + EX and AP + SED groups showed a downward trend. The above results suggest that aerobic exercise can upregulate the level of AdipoR1 in the AD mouse brain.

### 2.7. Exercise Promotes the Expression and Phosphorylation of AMPK in the Brain Cells of AD Mice

**Figure 7:**
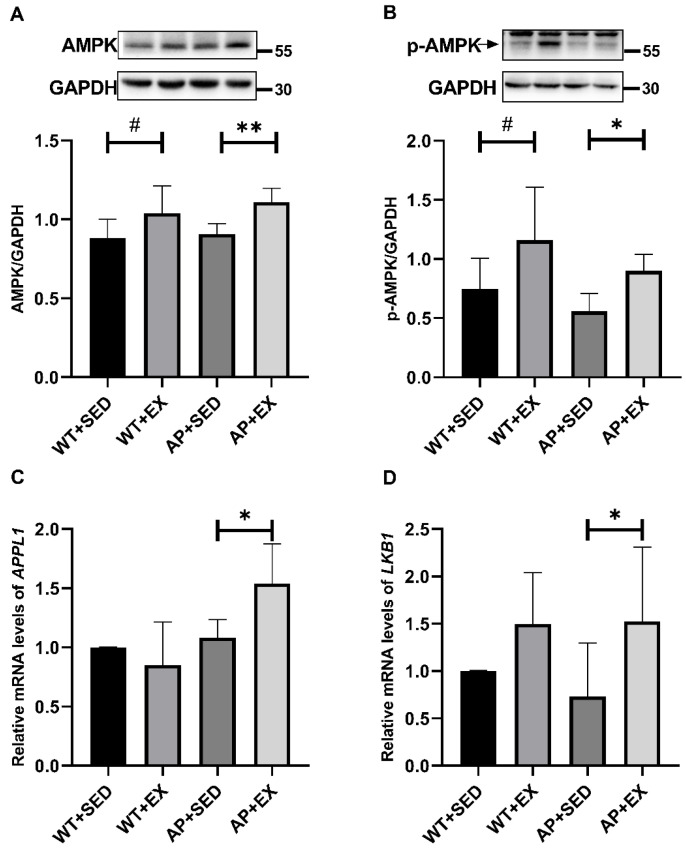
Exercise enhances AMPK activity in mouse brain tissue. Relative protein levels of (**A**) AMPK and (**B**) p-AMPK in brain tissue (*n* = 6). The relative expression levels of (**C**) *APPL1* and (**D**) *LKB1* mRNA in brain tissue were measured by RT-PCR (*n* = 5). Data are presented as means ± SDs. # *p* < 0.05 vs. WT + SED mice, * *p* < 0.05, and ** *p* < 0.01 vs. AP + SED mice.

Exercise upregulates the AdipoR1 levels in AD mouse brains. In order to further study the molecular mechanism by which the AdipoR1 signaling pathway participated in aerobic exercise’s alleviation of AD-like lesions, our group detected the expression and phosphorylation of the AMPK protein, a signaling molecule downstream of AdipoR1, and the mRNA expression levels of the signaling molecules between AdipoR1 and AMPK in mouse brain tissue: adaptor protein containing pleckstrin homology domain, phosphotyrosine-binding domain, and leucine zipper motif (*APPL1*) and liver kinase B1 (*LKB1*). As shown in Figure 7A,B, compared with the mice in the WT + SED group, aerobic exercise significantly increased the levels of the AMPK and p-AMPK proteins in the brains of the mice in the WT + EX group (*p* < 0.05). The level of p-AMPK in the brain cells of the mice in the AP + SED group showed a downward trend. Compared with the mice in the AP + SED group, aerobic exercise significantly increased the protein levels of AMPK and p-AMPK in the brains of the mice in the AP + EX group (AMPK, *p* < 0.01; p-AMPK, *p* < 0.05). The APPL1/LKB1 signaling pathway mediates the activation of AMPK by AdipoR1 [30,31,32]. As shown in Figure 7C,D, compared with the mice in the AP + SED group, aerobic exercise significantly increased the *APPL1* and *LKB1* mRNA expression levels in the brain cells of mice in the AP + EX group (*p* < 0.05). The above results suggest that aerobic exercise promotes the expression and activation of the downstream signaling molecule AMPK to a certain extent through AdipoR1 in the brain cells of APP/PS1 mice.

### 2.8. Exercise Activates TFEB and Lysosome Function in the Brain Cells of AD Mice

**Figure 8:**
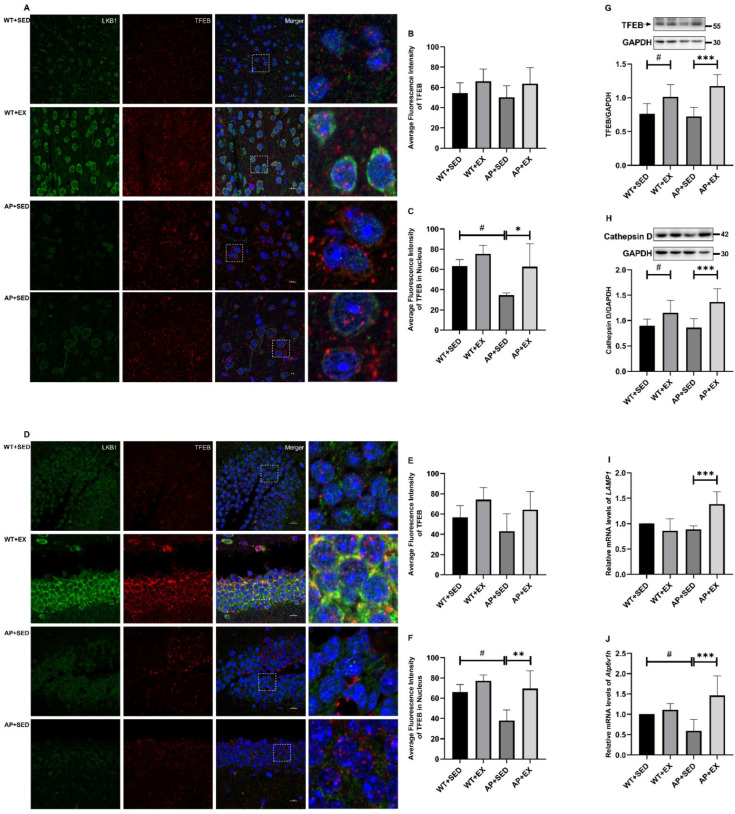
Exercise-activated TFEB enhances lysosomal function in brain cells. (**A**,**D**) Representative images of double immunofluorescence in mouse cortex and hippocampus (The green fluorescence is LKB1; the red fluorescence is TFEB; the blue fluorescence is the nucleus; the white dashed-square box is the area of the enlarged image). Scale bar: 10 μm. *n* = 3 per group. (**B**,**C**,**E**,**F**) Histograms showing the mean fluorescence intensity of TFEB and intranuclear TFEB in the cortex and hippocampus ((**B**) TFEB in cortex; (**C**) TFEB in the nucleus of the cerebral cortex; (**E**) TFEB in the hippocampus; (**F**) TFEB in the nucleus of the hippocampus). Relative protein levels of (**G**) TFEB and (**H**) cathepsin D in brain tissue (*n* = 6). Relative expression levels of (**I**) *LAMP1* and (**J**) *Atp6v1h* mRNA in brain tissue (*n* = 5). Data are presented as means ± SDs. # *p* < 0.05 vs. WT + SED mice, * *p* < 0.05, ** *p* < 0.01, and *** *p* < 0.001 vs. AP + SED mice.

Exercise upregulates the AdipoR1 levels in AD mice brain. AdipoR agonists have been reported to promote TFEB’s nuclear translocation and the expression of TFEB-controlled autophagic genes, such as cathepsin D, lysosomal-associated membrane protein 1 (LAMP1), and ATPase H+ transporting V1 subunit H (Atp6v1h), thereby enhancing lysosomal function and autophagic flux [33,34,35]. It is suggested that aerobic exercise may promote the nuclear translocation of TFEB in the brain cells of APP/PS1 mice by upregulating the AdipoR1 signaling pathway. Therefore, our group detected the expression of TFEB in mouse brain tissue, its nuclear translocation level, and the expression of lysosomal-function-related proteins and genes. As shown in Figure 8B,E, there was no significant difference in the mean fluorescence intensity of TFEB in the cortices and hippocampi of the mice in each group (*p* > 0.05), but aerobic exercise induced TFEB in the cortices and hippocampi of the mice in the WT + EX and AP + EX groups. The mean fluorescence intensity showed an upward trend. As shown in Figure 8C,F, compared with the mice in the WT + SED group, the mean fluorescence intensity of TFEB in the cortices and hippocampal nuclei of the mice in the AP + SED group was significantly decreased (*p* < 0.05). Compared with the mice in the AP + SED group, aerobic exercise significantly increased the mean fluorescence intensity of TFEB in the cortices and hippocampal nuclei of the mice in the AP + EX group (cortex, *p* < 0.05; hippocampus, *p* < 0.01). As shown in Figure 8G,H, compared with the mice in the WT + SED group, the protein levels of TFEB and cathepsin D in the brain cells of the mice in the WT + EX group were significantly increased (*p* < 0.05). Compared with mice in the AP + SED group, aerobic exercise significantly increased the protein levels of TFEB and cathepsin D in the brain cells of the mice in the AP + EX group (*p* < 0.001). As shown in Figure 8J, compared with the mice in the WT + SED group, the *Atp6v1h* mRNA expression levels of the mice in the AP + SED group were significantly downregulated (*p* < 0.05). As shown in Figure 8I,J, compared with the mice in the AP + SED group, aerobic exercise significantly increased the expression levels of the *LAMP1* and *Atp6v1h* mRNA in the brain cells of the mice in the AP + EX group (*p* < 0.001). The above results suggest that aerobic exercise can enhance the lysosomal function of APP/PS1 mouse brain cells by activating TFEB activity, which may be mediated by the AdipoR1 signaling.

### 2.9. Exercise Activated PLC/Kcnip3/Mid1/PP2A Signaling Pathway in the Brain Cells of AD Mice

**Figure 9:**
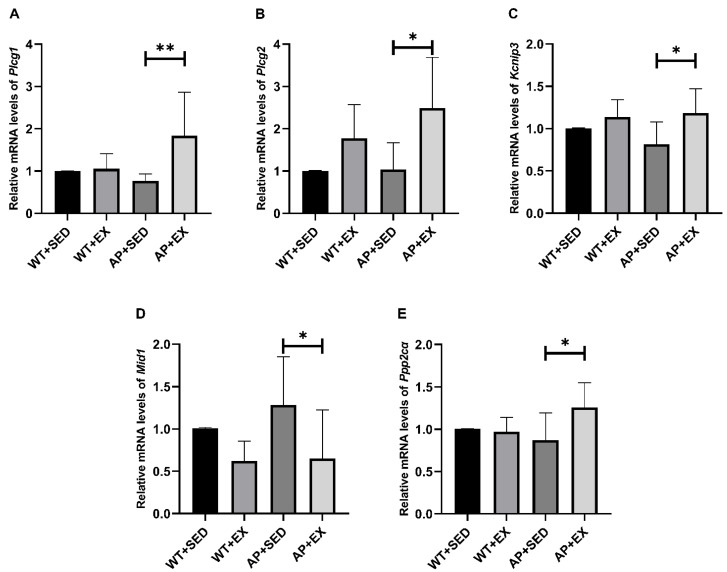
Effects of exercise on the *PLC*/*Kcnip3*/*Mid1*/*PP2A* signaling pathway in the brains of AD mice. The relative expression levels of the (**A**) *Plcg1*, (**B**) *Plcg2*, (**C**) *Kcnip3*, (**D**) *Mid1*, and (**E**) *Ppp2cα* mRNA in mouse brain tissue were measured by RT-PCR (*n* = 5). Data are presented as means ± SDs. * *p* < 0.05 and ** *p* < 0.01 vs. AP + SED mice.

To further study the possible molecular mechanism underlying the AdipoR1-mediated promotion of TFEB nuclear translocation in APP/PS1 mice by aerobic exercise, our group detected the mRNA expression levels of *PLC*/*Kcnip3*/*Mid1*/*PP2A*, involved in the upstream signaling pathway of TFEB [34]. As shown in Figure 9A–C,E, compared with the mice in the AP + SED group, the mRNA expression levels of phospholipase C gamma (*Plcg*) 1/2, *Kcnip3*, and protein phosphatase 2 catalytic subunit alpha (*Ppp2cα*) in the brain cells of the mice in the AP + EX group were all increased (*Plcg1*, *p* < 0.01; *Plcg2*, *p* < 0.05; *Kcnip3*, *p* < 0.05; *Ppp2cα*, *p* < 0.05). As shown in Figure 9D, compared with the mice in the AP + SED group, the expression level of the *Mid1* mRNA in the brain cells of the mice in the AP + EX group was significantly downregulated (*p* < 0.05). The above studies suggest that aerobic exercise activates the *PLC*/*Kcnip3*/*Mid1*/*PP2A* signaling pathway in the brain cells of APP/PS1 mice.

## 3. Discussion

Exercise alleviates AD-like lesions and cognitive impairment in APP/PS1 mice. Previous studies have shown that Aβ in the brain is the main cause of neuronal apoptosis and cognitive impairment in AD [1]. Endurance exercise can delay brain aging, preserve memory and cognitive function, and improve the symptoms of neurodegenerative diseases such as AD [36]. Studies have shown that aerobic exercise can effectively downregulate the expression of Bax and caspase3 in the brain of APP/PS1 mice, upregulate the expression of Bcl-2, and upregulate the ratio of Bcl-2/Bax, thereby inhibiting neuronal apoptosis and improving cognitive function [4,37]. Aerobic exercise increased the number of dendritic spines in APP/PS1 mice and was significantly associated with improved cognitive function [38]. Consistently, the results of this study showed that APP/PS1 mice had severe Aβ deposition in the brain, a loss of dendritic spines, and impaired spatial learning and memory, suggesting that the AD-model mice successfully developed AD-like lesions, while aerobic exercise reversed the above changes; inhibited Aβ deposition, brain cell apoptosis, and dendritic spine loss; and alleviated the spatial learning and memory impairment in APP/PS1 mice.

Autophagy, a catabolic pathway that maintains cellular metabolic balance and realizes cellular self-renewal, involves the autophagosome: a double-layer membrane shed by the intracellular rough endoplasmic reticulum that coats cellular components such as excessively accumulated proteins and damaged organelles in the cytoplasm and then fuses with a lysosome to form an autophagolysosome for degradation [23]. Autophagy is fundamentally active and efficient in normal neurons, whereas autophagy dysfunction occurs in AD [23,39]. Recent studies have shown that dysfunction of the autophagy–lysosomal pathway in AD precedes AD-related pathologies such as Aβ deposition and induces neuronal apoptosis as well as extracellular plaque formation and expansion [40]. It is suggested that abnormalities in autophagy not only prevent toxic proteins such as Aβ being removed, but also aggravate AD-like lesions [2,23,39]. The results of this study show that the expression of the *mTOR* gene was upregulated and that *Beclin1* and the LC3-II/I ratio was downregulated in APP/PS1 mice, suggesting that autophagic activity was inhibited; at the same time, the expression of the *P62* mRNA did not change and even showed a downward trend, while its protein expression was upregulated, suggesting that autophagic degradation was hindered. The above studies suggest that abnormal autophagy in AD is an important factor in aggravating the disease.

Normal autophagy not only contributes to the degradation of toxic proteins such as Aβ but is also critical for maintaining dendritic spine structure and spine pruning [2,23,24]. It was found that the attenuation of dynamin-related protein 1 gene expression caused autophagy activation, increased dendritic spines, and enhanced cognitive function in transgenic Tau mice [41]. The results of this study show that aerobic exercise reversed the above abnormal changes in autophagy, downregulated *mTOR* gene expression, upregulated *Beclin1* and the LC3-II/I ratio, upregulated the *P62* mRNA expression level, and downregulated the P62 protein level. It is suggested that aerobic exercise alleviates AD-like lesions, such as the loss of dendritic spines and cognitive dysfunction, by alleviating abnormal autophagy in AD brain cells. Previous studies have also shown that enhanced autophagy plays an important role in inhibiting Aβ levels and neural apoptosis [23]. Exercise can promote the degradation of Aβand inhibit neural apoptosis by regulating autophagy in AD brain cells, thereby reducing the Aβ plaques and alleviating cognitive dysfunction [4,5,6]. However, the specific pathway through which exercise regulates AD autophagy remains unclear.

APN plays an important role in the beneficial effects of physical exercise in protecting brain tissue structure and improving cognitive function [18]. Numerous studies have found that APN knockout abolishes the beneficial effects of physical exercise on hippocampal neurogenesis and cognitive function in mice with chronic disease [11,12,13,14]. It is suggested that exercise can alleviate the damage to brain structure and function caused by chronic diseases through APN. APN plays an important role in relieving AD [7,15,16,17,42]. Studies have shown that endogenous APN deficiency causes a decrease in dendritic spine density in mouse neurons, while exogenous APN treatment increases dendritic spine density [17,42]. Consistent with this study, aerobic exercise alleviated the loss of dendritic spines. It is suggested that aerobic exercise may alleviate the loss of dendritic spines through APN. APN treatment alleviated spatial learning and memory impairment in 3xTg-AD mice by reducing neuroinflammation and the impairment of synaptic plasticity [15]. APN deficiency accelerates Aβ deposition and exacerbates learning and memory impairment; treatment with the APN agonist AdipoRon alleviates Aβ plaque deposition in APP/PS1 mice and cognitive impairment in 5xFAD*APN KO mice [16]. AD patients, APP/PS1 mice, and Adipo^−/−^ mice all showed decreased levels of AdipoR1 in brain tissue and impaired learning and memory functions, but treatment with an adiponectin-mimetic novel nonapeptide reversed the above changes [17]. It is suggested that exercise may relieve AD conditions through APN and is associated with AdipoR1. The alleviating effect of APN on AD is achieved, to a certain extent, through the activation of autophagy by AdipoR1. A study found that APN treatment caused the upregulation of AdipoR1 expression levels and the activation of autophagy in the brain tissue in global cerebral ischemia–reperfusion [43]. The APN agonist AdipoRon inhibits Aβ levels by activating autophagy while increasing AdipoR1 levels; similarly, the overexpression of AdipoR1 resulted in the upregulation of the autophagy-related protein LC3-II and the downregulation of Aβ levels in N2a/APPswe cells [16]. The results of this study show that aerobic exercise can significantly upregulate the expression of AdipoR1 in the brains of APP/PS1 mice, alleviate abnormal autophagy, and inhibit the level of Aβ plaques. In addition, exercise-induced downregulation of AdipoR1 in wild-type mice may be due to the negative feedback between APN and AdipoR1; under the pathological conditions of AD, aerobic exercise fails to upregulate APN levels, but is enhanced by compensatory upregulation of AdipoR1 levels APN biological effects [43]. However, the specific reasons need to be clarified in our future studies. Based on the above results, aerobic exercise is likely to regulate AD autophagy by upregulating the AdipoR1 levels and to relieve AD-like lesions and cognitive dysfunction in APP/PS1 mice. This view was further confirmed in the follow-up exploration of AdipoR1 downstream signaling molecules.

AMPK, a signaling molecule that acts downstream of AdipoR1, plays an important role in exercise’s regulation of autophagy in AD [6,30,44,45,46]. The previous study of our group showed that aerobic exercise activated autophagy by activating the AMPK/Beclin1 signaling pathway and alleviated the morphological and structural damage of the rat hippocampus [44,45]. Consistently, previous studies have found that long-term exercise induces enhanced AMPK activity in the brain tissue of AD mice, the activation of autophagy, and the remission of AD-like lesions [4,6,46]. AMPK mediates AdipoR1’s regulation of autophagy in AD and alleviation of the disease [16,17]. Studies have found that an adiponectin-mimetic novel nonapeptide enhances synaptic plasticity and memory function in AD and Adipo^−/−^ mice by activating the AdipoR1/AMPK signaling pathway [17]. AdipoR agonists upregulate autophagy and reduce Aβ levels in N2a/APPswe cells by activating the AdipoR1/AMPK/mTOR signaling pathway, while AMPK inhibitors reverse the above changes [16]. The results of this study show that aerobic exercise induced an upregulation of the mRNA expression of *APPL1*/*LKB1*, a messenger between AdipoR1 and AMPK; enhanced AMPK activity; decreased the mRNA expression of *mTOR*, a molecule acting downstream of AMPK, in the brain tissue of APP/PS1 mice; and alleviated autophagy abnormalities. In addition, the downward trend of *APPL1* mRNA expression in wild-type mice caused by exercise may be related to the increased APN levels and the downward trend of AdipoR1. Further clarification is required in future studies. Based on the above results, aerobic exercise is likely to alleviate autophagy abnormalities in the brain cells of APP/PS1 mice by activating the AdipoR1/AMPK signaling pathway.

In addition, this study found that the LC3-II/I ratio in the brain tissue of wild-type mice was downregulated by aerobic exercise, and the expression of the P62 protein showed a downward trend, but the expression of the *P62* mRNA showed an upward trend. Consistently, related studies have shown that drug treatment downregulates cellular LC3-II, p62, and Aβ expression by enhancing lysosomal function, restores autophagic flux, and is associated with TFEB [47,48]. It is suggested that the downregulation of the LC3-II /I ratio and P62 protein level in the brains of wild-type mice and the decrease in the P62 protein levels in the brains of AD mice caused by aerobic exercise may be due to the enhanced lysosomal function that promotes autophagic degradation.

The enhancement of lysosomal function plays an important role in exercise’s enhancement of autophagy in AD. Studies have shown that decreased lysosomal acidification accompanied by impaired autophagic substrate clearance is an important factor in the induction of AD-related pathologies such as Aβ deposition [40]. TFEB regulates the expression of lysosomal-function-related genes such as *LAMP1* and *cathepsin D* and autophagy-related genes, thereby enhancing the autophagy–lysosomal pathway, reducing neural apoptosis, and exerting neuroprotective effects [35,49]. Drug treatment enhances the autophagolysosomal pathway by activating TFEB, thereby promoting the degradation of toxic proteins in AD models, and PP2A plays an important role in promoting TFEB’s nuclear translocation [33]. The results of this study show that aerobic exercise induced enhanced TFEB protein expression and nuclear translocation in APP/PS1 mice; upregulated the expression of lysosomal-function-related molecules such as cathepsin D, *LAMP1*, and *Atp6V1h*; enhanced P62 protein degradation; and alleviated autophagy abnormalities. In addition, the level of *LAMP1* mRNA in wild-type mice did not change significantly or even show a downward trend after exercise intervention, which may be related to the downregulation of LC3-II/I ratio and the enhancement of lysosomal function. Further clarification is required in future studies. It is suggested that aerobic exercise enhances lysosomal function by promoting TFEB protein expression and nuclear translocation in APP/PS1 mice, thereby alleviating autophagy abnormalities. Previous studies have also shown that exercise can enhance the expression of lysosomal-function-related proteins such as cathepsin D/L and LAMP1 in AD brain tissue by activating the AMPK/TFEB signaling pathway, improve autophagic degradation, and promote autophagy, thereby promoting Aβ degradation, inhibiting apoptosis, and reducing Aβ plaque [4,6,50]. However, the AMPK/SIRT1 signaling pathway mainly mediates the enhancement of TFEB gene transcription by exercise without affecting the nuclear translocation of TFEB [50]. Therefore, how exercise promotes TFEB nuclear translocation in AD brain cells is unclear [6,50].

It has been reported that both APN-KO and 5xFAD*APN KO mice have decreased lysosomal-function-related proteins such as LAMP1 and cathepsin D [16]. AdipoR agonists promote intracellular TFEB nuclear translocation and TFEB-controlled autophagic gene expression [35]. The results of this study show that aerobic exercise upregulates the AdipoR1 levels in the brain cells of APP/PS1 mice and enhances TFEB’s nuclear translocation and lysosomal function. It is suggested that aerobic exercise may promote the nuclear translocation of TFEB in the brain cells of APP/PS1 mice by upregulating the AdipoR1 levels, thereby enhancing lysosomal function and alleviating abnormal autophagy. This view is explored further below.

Our group preliminarily explored the possible molecular mechanism by which aerobic exercise promotes the nuclear translocation of TFEB in AD brain cells by upregulating AdipoR1 levels. First, AdipoR1 regulates intracellular Ca^2+^ levels by activating PLC [30,31,35,51]. Studies have shown that PLC-mediated APN promotes the release of Ca^2+^ from the endoplasmic reticulum, which is not affected by extracellular Ca^2+^ [30]. Further studies found that AdipoR agonists were dependent on intracellular Ca^2+^ for their promotion of TFEB’s nuclear translocation, independent of extracellular Ca^2+^, AMPK, protein kinase B (Akt), extracellular signal-regulated kinase (ERK) 1/2, and mTOR [35]. It was also found that the inhibition of AdipoR1 expression abolished the increase in Ca^2+^ levels caused by APN [31]. It is suggested that APN regulates intracellular Ca^2+^ mainly through AdipoR1, but whether AdipoR1 regulates Ca^2+^ in terms of intracellular release or extracellular influx remains controversial, and there may be differences in different cells [30,31,51]. It is suggested that the AdipoR1/PLC signaling pathway plays an important role in promoting the nuclear translocation of TFEB. Second, the regulation of intracellular Ca^2+^ by PLC can activate the Kcnip3/Mid1/PP2A signaling pathway, thereby promoting the nuclear translocation of TFEB [34,52,53,54]. Studies have shown that Ca^2+^ inhibits *Mid1* transcription by activating Kcnip3, thereby promoting PP2A stabilization and enhancing TFEB’s nuclear translocation and the autophagy–lysosomal pathway [34]. AD-related studies have also shown that Mid1 is significantly increased in the brains of AD patients, and that the inhibition of Mid1 expression can enhance the stability of PP2A and promote Tau dephosphorylation [52]. PP2A activity was reduced in APP/PS1 transgenic mice, and the reactivation of PP2A activity effectively suppressed the levels of toxic proteins such as Aβ and protected neurons and synapses, thus alleviating cognitive dysfunction [53]. In a series of human and murine cell experiments, PP2A, similar to APN, dephosphorylated TFEB independently of mTOR and promoted the nuclear translocation of TFEB [54]. Our group hypothesized that the AdipoR1 might promote TFEB’s nuclear translocation in AD brain cells by regulating the *PLC*/*Kcnip3*/*Mid1*/*PP2A* signaling pathway. We found that aerobic exercise upregulated the AdipoR1 levels in the brain cells of APP/PS1 mice. Based on the above, our research group explored the possibility that aerobic exercise affected the mRNA expression of *PLC*/*Kcnip3*/*Mid1*/*Ppp2cα* in AD mouse brain cells. We found, in this study, that the intervention of aerobic exercise resulted in increased mRNA expression of *Plcg1/2*, *Kcnip3*, and *Ppp2cα* in the brain cells of APP/PS1 mice, while the expression of the *Mid1* mRNA was inhibited. In addition, in this study, the mRNA expression levels of *PLC*/*Kcnip3*/*Mid1*/*Ppp2cα* and other molecules in wild-type mice did not change significantly after exercise intervention, which may be related to their healthy and stable internal environment effectively alleviating stress responses. Further clarification is required in future studies. Based on the above results, the AdipoR1/PLC/PP2A signaling pathway may play an important role in aerobic exercise’s promotion of TFEB’s nuclear translocation and enhancement of the autophagy–lysosomal pathway in the brain cells of AD mice. This research group will further explore and demonstrate this in future research.

## 4. Materials and Methods

### 4.1. Experimental Animals and Grouping

Twenty 6-week-old male wild-type mice, whose strain number is C57BL/6J, were purchased from Changsha Tianqin Biotechnology Co., Ltd. (Changsha, China, license number: SCXK (Xiang) 2019-0014). Twenty 8-week-old male APP/PS1 double-transgenic mice were purchased from Changzhou Cavens Laboratory Animal Co., Ltd. (Changzhou, China, license number: SCXK (Su) 2016-0010). All 40 mice were reared in the animal room of the Hunan Provincial Key Laboratory of Physical Fitness and Exercise Rehabilitation according to the national standard rodent-feeding program. The ambient temperature was kept at 24 °C, and sufficient water and an appropriate amount of experimental animal feed (GB14924.3-2010, China) were provided. Animals strictly abided by the 3R principles during feeding and experimentation, and the regulations of the ethics committee of the unit were followed (Ethics Section 2021 No. 198).

Twenty wild-type (WT) mice were randomly divided into a sedentary (SED) group (WT + SED) and an exercise (EX) group (WT + EX), with 10 mice in each group. Twenty APP/PS1 double-transgenic (AP) mice were randomly divided into a sedentary (SED) group (AP + SED) and an exercise (EX) group (AP + EX), with 10 mice in each group.

### 4.2. Exercise Protocols

**Figure 10:**
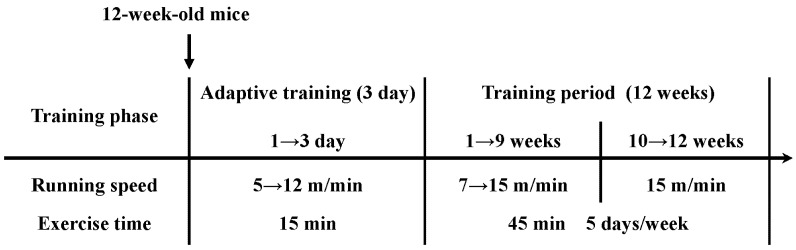
Aerobic exercise protocol for mice.

The mouse exercise training device (patent number: ZH-PT/5S) was selected for the experiment. The PT-type mouse animal experimental treadmill (flat treadmill) is mainly used for the running exercise training of small mice. Referring to relevant materials and combining them with the needs of this experiment, a regular aerobic exercise model was established [4,55]. The exercise program was as follows (Figure 10):

After the mice were raised to 12 weeks of age, the mice in the WT + EX and AP + EX groups underwent adaptive training for three days, exercising for 15 min every day at speeds of 5, 8, and 12 m/min, increasing day by day. After that, random group numbering was carried out. Formal exercise occurred at 7, 8, 9, 10, 11, 12, 13, 14, and 15 m/min weekly increments from 1 to 9 weeks, and subsequently at a constant speed for three weeks, with a total of 12 weeks of exercise. Training occurred on every day of the week, except Thursday and Sunday. The training time was from 16:00 to 19:00, and the exercise periods lasted for 45 min.

### 4.3. Morris Water Maze Test

To assess the learning and memory abilities of the mice, the test was performed as previously described [4,11]. Mice were tested in a circular pool with a diameter of 1.5 m and a height of 0.5 m. One day before the test, the mice were acclimated in the pool for 10 min. During the learning phase, the platform (10 cm in diameter) was immersed 1.5 cm below the water surface, keeping the water temperature around 25 °C, adding a nontoxic white dye to make the water opaque and hide the platform. During the learning phase, each mouse performed 4 trials per day, which were pseudo-randomly selected in the four quadrants, and the quadrant where the platform was located was the target quadrant; this lasted a total of 5 days. Each mouse had 60 s to locate the hidden platform. If it failed to reach the hidden platform within 60 s, it was guided to the platform, and 10 s stop learning was conducted. During the retention phase, the platform was removed, and the mice swam freely for 60 s. All the trials were video-recorded, and the data were analyzed using the Xeye Aba animal behavior video analysis system (Beijing Tianming Hongyuan Technology Development Co., Ltd., Beijing, China).

Twenty-four hours after the water maze test, the mice in each group were fasted for 12 h and then anesthetized with isoflurane. Serum was collected for determining the biochemical indices. After the heart was perfused with normal saline, the left hemispheres of 3 mice in each group were fixed in 4% paraformaldehyde and then stored in a solution containing 0.02% sodium azide for immunohistochemical experiments. After the rest of the mice in each group were anesthetized and sacrificed, the brains were quickly removed, frozen in liquid nitrogen, and stored at −80 °C.

### 4.4. Immunohistochemical Detection

Fixed hemibrains were cut into 50 μm coronal sections using a LEICA VT1000S vibratome and stored in a solution containing 0.02% sodium azide. After they were washed with TBS, they were soaked in a 3% hydrogen peroxide methanol solution for 30 min. After TBS cleaning, they were then soaked in 88% formic acid for 7 min, TBS-A (1 L of TBS + 1 mL of Tritonx-100) for 15 min, and TBS-B (100 mL of TBS-A + 2 g of BSA) for 30 min. The sections were incubated with a primary antibody (1:1000, 6E10-Mouse IgG, Biolegend, San Diego, CA, USA) overnight at 4 °C. After they were washed with TBS-A/B, they were incubated with a secondary antibody for 1 h at room temperature. After they were washed with TBS-A, they were incubated in a solution prepared using the ABC Kit (Vector, Tokyo, Japan, Cat: PK-4002) at room temperature for 1 h. After they were washed with TBS, they were soaked in a DAB solution for 2 min 30 s, and the staining was observed at ×40 magnification. The Image-Pro Plus 6.0 software (Media Cybernetics, Inc., Rockville, MD, USA) was used to analyze the percentages of Aβ plaque areas in the field of view of each brain slice.

### 4.5. Enzyme-Linked Immunosorbent Assay

The content of APN protein in the serum and brain tissue was detected according to the instructions of the mouse adiponectin ELISA kit (Quanzhou Ruixin Biotechnology, Cat: RX202420M, Quanzhou, China).

### 4.6. Real-Time PCR

Total RNA from brain tissue was extracted with TRIzol (Ambion, Cat: 15596026, Austin, TX, USA) and reverse-transcribed into cDNA using TransScript^®^ One-Step gDNA Removal and cDNA Synthesis SuperMix (TransGen Biotech, Cat: AT311-02, Beijing, China). qRT-PCR was performed using the 2×SYBR Green qPCR Master Mix (Servicebio, Cat: G3320, Wuhan, China) and the CFX Connect™ Real-Time System (Bio-Rad, Singapore). Using GAPDH as the internal reference gene, the relative expression of the target gene was calculated by the 2^−∆∆CT^ method. The sequences of the primers used for the qRT-PCR were as follows (Table 1).

### 4.7. Western Blotting

An RIPA (Servicebio, Cat: G2002-100 ML, Wuhan, China) lysis buffer was used to extract the total protein from brain tissue, and various protease inhibitors (Servicebio, Cat: G2006-250UL, G2007-1ML, and G2008-1ML, Wuhan, China) and a 2X protein loading buffer (Servicebio, Cat: G2031-1ML, Wuhan, China) were added. The mixture was heated at 95 °C for 10 min and stored at −20 °C after aliquoting. After gel preparation was completed using an SDS-PAGE gel rapid preparation kit (Servicebio, Cat: G2037, Wuhan, China), the protein lysates were separated by electrophoresis and transferred to a 0.45 μm PVDF membrane (Servicebio, Cat: G6015-0.45, Wuhan, China), with 5% skim milk used for blocking. The membrane was incubated with a primary antibody at 4 °C overnight, and then with a secondary antibody at room temperature for 1 h. Visualization was performed using an ECL kit (Servicebio, Cat: G2014-50 mL, Wuhan, China), and quantification was performed using the ImageJ 1.53q software (Wayne Rasband and contributors National Institutes of Health, Bethesda, MD, USA).

The primary antibodies were as follows: AdipoR1 (Abcam, Cat: ab126611, 1:2500, Cambridge, UK), Bax (Proteintech, Cat: 60267-1-Ig, 1: 1000, Chicago, IL, USA), Bcl-2 (Proteintech, Cat: 26593-1-AP, 1:1000, Chicago, IL, USA), AMPK (Abcam, Cat: ab32047, 1:2500, Cambridge, UK), p-AMPK (Abcam, Cat: ab133448, 1:2500, Cambridge, UK), LC3-II (Proteintech, Cat: 18725-1-AP, 1: 1000, Chicago, USA), TFEB (Proteintech, Cat: 13372-1-AP, 1: 2000, Chicago, IL, USA), LKB1 (Servicebio, Cat: GB111236, 1: 1000, Wuhan, China), P62 (Abcam, Cat: 109012, 1: 2500, Cambridge, UK), cathepsin D (Abcam, Cat:ab75852, 1:2500, Cambridge, UK), and GAPDH (Servicebio, Cat:GB12002, 1:1000, Wuhan, China).

### 4.8. Golgi Staining

A Golgi Stain Kit (FD Rapid Golgi Stain Kit PK-401, FD Neuro Technologies, Columbia, MD, USA) was used according to the manufacturer’s instructions. The left hemibrains of 4 mice in each group were placed into a mixture of Golgi staining A/B (1:1) and stored at room temperature in the dark for 24 h. They were then placed into a fresh mixture and stored for another 2 weeks. The hemibrains were then put into Solution C and stored in the dark for 12 h. Solution C was then refreshed, and the specimens were stored for another 72 h. The brain tissue was cut into 200 μm coronal sections with a LEICA VT1000S vibrating microtome, and the sections were placed on a gelatin-coated glass slide and dried naturally at room temperature for 1–3 days in the dark. The slides with the sections were washed with double-distilled water for 2 × 3 min and then stained in a staining tank for 10 min (Solution D:Solution E:double-distilled water = 5:5:15 mL). After they were washed with double-distilled water for 2 × 4 min, they were dehydrated using 50%, 75%, and 95% ethanol for 5 min and using anhydrous ethanol for 3 × 5 min, made transparent with a xylene solution for 2 × 5 min, sealed with neutral resin, and air-dried at room temperature. Images were acquired at 1000× magnification with 5–10 neurons each in the cortex and hippocampus of each sample (*n* = 3–4), with 3–5 segments per neuron, apical and basal. The Image J 1.53q software (Wayne Rasband and contributors National Institutes of Health, USA) was used to calculate the exact length. Dendritic segments larger than 30 μm were selected, and the number of spines on the corresponding dendritic segment was counted. The density of the dendritic spines is expressed as the number of dendritic spines/10 μm [56].

### 4.9. Immunofluorescence Staining

The fixed hemibrains were embedded in paraffin, placed in a paraffin microtome, and cut into 5 μm coronal sections. After the sections were deparaffinized and rehydrated, they were placed in an EDTA antigen retrieval buffer (Servicebio, Cat: G1206, Wuhan, China) for antigen retrieval in a microwave oven (8 min at medium heat, 8 min at intervals, and 7 min at medium-low heat) and then washed with PBS (pH 7.4) for 3 × 5 min. They were then blocked with 3% hydrogen peroxide for 25 min at room temperature and washed with PBS. A 3% BSA solution (Servicebio, Cat: G5001, Wuhan, China) was used to block them for 30 min. The primary antibody was then added and incubated with the sections overnight at 4 °C. After washing with PBS, the corresponding secondary antibody was added and incubated with the sections at room temperature for 50 min. After washing with PBS, CY3-TSA (Servicebio, Cat: G1223, Wuhan, China) or FITC-TSA (Servicebio, Cat: G1222, Wuhan, China) was added, and the mixture was shaken and washed on a destaining shaker (for the second primary antibody, this was repeated, but hydrogen peroxide blocking was avoided). A DAPI staining solution (Servicebio, Cat: G1012, Wuhan, China) was added and incubated with the sections at room temperature for 10 min in the dark. Images at 1000× magnification were acquired using a confocal laser microscope (NIKON Eclipse Ti, Nikon, Tokyo, Japan). The Image J 1.53q software (Wayne Rasband and contributors National Institutes of Health, USA) was used to calculate the average immunofluorescence intensity of the target area, and the threshold was set by the default algorithm.

### 4.10. Statistical Analysis

The obtained data were statistically analyzed and graphed using the GraphPad Prism 8.0.1 software (GraphPad Software, Inc., San Diego, CA, USA), and the data are expressed as the means ± standard deviations (SDs). A Student’s *t*-test or one-way ANOVA was used, and the LSD method was used for post hoc comparisons to test the significance of differences between data groups. *p* < 0.05 indicated a significant difference, *p* < 0.01 indicated a very significant difference, and *p* < 0.001 indicated an extremely significant difference.

## 5. Conclusions

**Figure 11:**
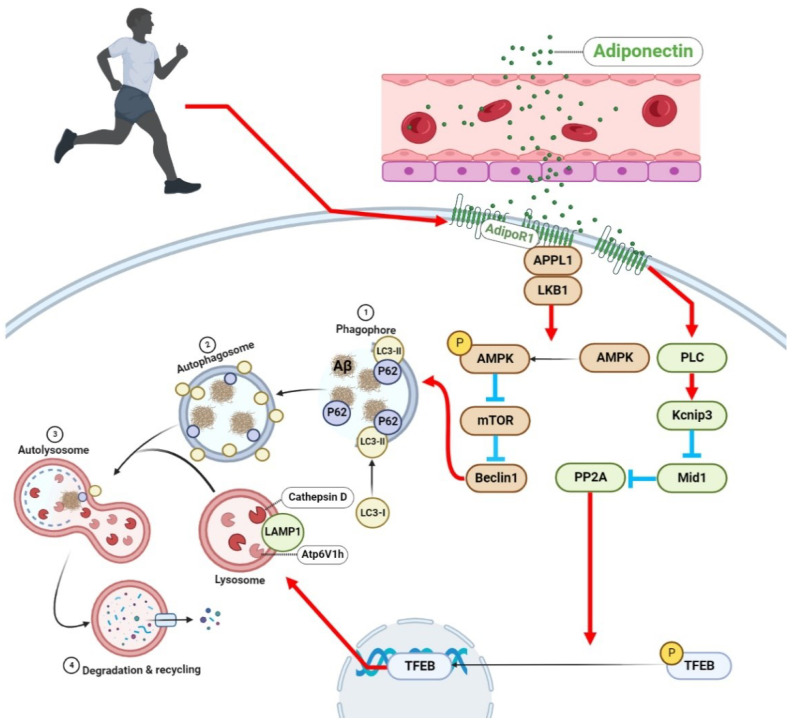
Proposed model for aerobic exercise’s alleviation of abnormal autophagy in brain cells of APP/PS1 mice by upregulating the AdipoR1 levels (created with BioRender.com). Available online: https://app.biorender.com/illustrations/6279e002dbc8411b3f12fe3f (accessed on 22 July 2022) Aerobic exercise upregulates AMPK and TFEB activity by upregulating the AdipoR1 levels in the brains of APP/PS1 mice, enhances lysosomal function, and improves abnormal autophagy, thereby alleviating AD-like lesions, Aβ deposition, and cognitive dysfunction.

This study was the first to explore the role of the APN–AdipoR1 signaling pathway in exercise’s alleviation of AD-like pathophysiological progression through regulating autophagy. The results of this study suggest that aerobic exercise may enhance the autophagy–lysosomal pathway through the activation of the AdipoR1/AMPK/TFEB signaling pathway in APP/PS1 mice and alleviate autophagy abnormalities, thereby reducing Aβ deposition, inhibiting neural apoptosis and dendritic spine loss, and alleviating cognitive dysfunction in AD (Figure 11). These findings suggest that the AdipoR1 plays an important role in aerobic exercise’s alleviation of abnormal autophagy in AD brain cells and alleviation of AD-like lesions.

The roles of the APN–AdipoR1 signaling pathway in exercise’s regulation of autophagy and the alleviation of AD are very complex. Whether it is the effect of different forms of exercise on the APN–AdipoR1 signaling pathway, or the effect and mechanism of the APN–AdipoR1 signaling pathway in AD autophagy abnormalities, there are still many gaps and deficiencies that need to be further studied. This provides new ideas for revealing the mechanism of exercise-regulated autophagy in AD and even the treatment of AD.

## Figures and Tables

**Table 1:** Sequences of primers for target genes.

Gene	Forward Primer	Reverse Primer
*AdipoR1*	AATGGGGCTCCTTCTGGTAAC	GCAGACCTTATACACGAACTCC
*APPL1*	AGCCAGTGACCCTTTATATCTGC	AGGTATCCAGCCTTTCGGGTT
*LKB1*	CTGGACTCCGAGACCTTATGC	CAAGCTGGATCACATTCCGAT
*mTOR*	CTTCTTCCGTTCTATCTC	AAGTGTCAATCTGTATGG
*Beclin1*	GAACTCTGGAGGTCTCGCT	CACCCAGGCTCGTTCTACC
*P62*	AGTCCAGAATTCCTGCCTGA	TTCATTCAACTTCACATGAA
*LAMP1*	CTGCTCCTGCTGCTGCT	AATTGTGAGGCTGGG
*Atp6v1h*	CCAAGATGGACATTCGAGGTG	CACTTTGTTGGCACGAACTTC
*Plcg1*	GAGACGCGCCAGATCACAT	AAAGTCCCGAGAAGTCTTCCC
*Plcg2*	AAATCCGTCCGGGGAAGAAC	TCCTCCTTTGAGTCCGTTGC
*Kcnip3*	AGTGAACTGGAGTTATCCACGG	GTGAACTTGGTCTGAGCTTGT
*Mid1*	CTGTGACGGCACCTGTCTC	AAACGGCTGACTGTTGGTCTT
*Ppp2cα*	ATGGACGAGAAGTTGTTCACC	CAGTGACTGGACATCGAACCT
*GAPDH*	CATGGCCTTCCGTGTTCCTA	CCTGCTTCACCACCTTCTTGAT

## Data Availability

The data contained within the paper are available from the authors upon request.
